# Suppression of MCP-1, IFN-γ and IL-6 production of HNSCC *ex vivo* by pembrolizumab added to docetaxel and cisplatin (TP) exceeding those of TP alone is linked to improved survival

**DOI:** 10.3389/fimmu.2024.1473897

**Published:** 2025-01-15

**Authors:** Jana Wellhausen, Louisa Röhl, Michael Berszin, Irene Krücken, Veit Zebralla, Markus Pirlich, Matthaeus Stoehr, Susanne Wiegand, Andreas Dietz, Theresa Wald, Gunnar Wichmann

**Affiliations:** ^1^ Department of Otorhinolaryngology, Head and Neck surgery, University Hospital Leipzig, Leipzig, Germany; ^2^ Institute of Pathology, University Hospital Leipzig, Leipzig, Germany; ^3^ The Comprehensive Cancer Center Central Germany, Leipzig University Hospital, Leipzig, Germany; ^4^ Department of Otorhinolaryngology, Head and Neck Surgery, Christian-Albrechts-University Kiel, Kiel, Germany

**Keywords:** PD-1:PD-L1 immune-checkpoint inhibitor (ICI) pembrolizumab, local and locoregional advanced head and neck squamous cell carcinoma (HNSCC), neoadjuvant (induction) chemotherapy, predictive assay for chemoresponse-evaluation, biomarker research, monocyte chemoattractant protein 1 (MCP-1, CCL2)

## Abstract

**Background:**

Adding pembrolizumab, an anti-PD-1 antibody approved for treatment of head and neck squamous cell carcinoma (HNSCC) to neoadjuvant (induction-) chemotherapy utilizing docetaxel and cisplatin (TP) followed by radiotherapy may improve outcome in larynx organ-preservation (LOP) that is investigated in the European Larynx-Organ preservation Study (ELOS). As biomarkers for response to TP and pembrolizumab +TP are missing but may include cytokines, this work aims on determining cytokines potentially linked to outcome as prognostic markers sufficient to predict and/or monitor response to successful LOP.

**Methods:**

Collagenase IV digests were generated from 47 histopathological confirmed HNSCC tumor samples and seeded in 96-well plates containing pembrolizumab, docetaxel, cisplatin either solely or in binary or ternary combination. According to the FLAVINO protocol, supernatants were collected after 3 days, adherent cells fixed using ethanol, air-dried and pan-cytokeratin positive epithelial cells counted using fluorescence microscopy. The cytokines IL-6, IL-8, IFN-γ, IP-10, MCP-1, TNF-α, and VEGF in the supernatant were quantified by sandwich ELISA.

**Results:**

The mode of interaction between pembrolizumab and TP was assessed and correlated to outcome (overall, disease-specific and progression-free survival of patients). Suppression of MCP-1, IFN-γ and IL-6 production by pembrolizumab + TP exceeding the suppressive effect of TP was detected in the majority of samples and linked to improved survival. Multivariate Cox proportional hazard regression modeling revealed MCP-1, IFN-γ and IL-6 as independent outcome predictors.

**Conclusions:**

Comparing response to TP vs. pembrolizumab vs. TP + pembrolizumab may allow for identification of patients with superior outcome independent from treatment applied.

## Introduction

Head and neck squamous cell carcinoma (HNSCC) are a group of cancer emerging from the epithelia of the upper aerodigestive tract. While early stages can be cured through monomodal therapies, either surgical resection or radiotherapy, local and locoregional advanced (LA) HNSCC can be cured in sufficiently high frequencies only by combining treatment modalities, for instance surgery followed by post-operative radiotherapy (Op+PORT) or radio-chemotherapy (Op+PORCT) or concurrent radio-chemotherapy (CRT). Ablative surgery can be very devastating for health-related quality of life (QoL), and larynx organ preservation (LOP) in LA laryngeal and hypopharyngeal squamous cell carcinoma (LA-LHSCC) that can only be surgically treated by total laryngectomy (TL) is therefore very desirable. Two alternative multimodal LOP approaches, either induction chemotherapy (IC) followed by radiotherapy (IC+RT) or platinum-based concurrent radio-chemotherapy (CRT), are possible LOP options. Although IC+RT is already recommended in the German guideline on diagnosis, treatment, and follow-up of laryngeal cancer for only by total laryngectomy resectable advanced laryngeal and/or hypopharyngeal cancer responding to IC (1), LOP through IC+RT still remains experimental and is furthermore investigated in clinical LOP trials (2–6). LOP approaches are often discussed as being potentially more harmful for the patient compared to most early TL (without neoadjuvant treatment) followed by either postoperative radiotherapy (TL+PORT) or platinum-based radio-chemotherapy (TL+PORCT). However, using propensity score (PS) matched analyses we recently demonstrated that IC+RT according to DeLOS-II utilizing IC with docetaxel and cisplatin (TP) improves overall (OS), disease-specific (DSS), and event-free survival (EFS) compared to TL+PORT, TL+PORCT and CRT (6). The aim of the ELOS trial is to go one step further by analyzing if addition of the immune-checkpoint inhibitor (ICI) pembrolizumab to TP results in even more improved LOP according to laryngectomy-free survival, increased OS and EFS of LA-LHSCC otherwise amenable for TL (7, 8).

In recent years, significant progress has been made in the treatment of R/M HNSCC with therapeutic approaches in the field of immune checkpoint inhibitors (ICI). The KEYNOTE-048 was a phase-III RCT comparing pembrolizumab monotherapy, pembrolizumab with chemotherapy (cisplatin and 5-fluorouracil), and cetuximab with chemotherapy (cisplatin and 5-fluorouracil) (ClinicalTrials.gov, number NCT02358031). The KEYNOTE-048 study led to the approval of pembrolizumab as monotherapy or in combination with chemotherapy with platinum and 5-fluorouracil (5-FU) for first-line treatment for PD-L1-positive R/M HNSCC with CPS ≥ 1 (9).

Immunomodulation by ICI targeting PD-1 has also been used in the curative setting, *e.g.* the RCT ADRISK (ClinicalTrials.gov NCT03480672 (10); or NadiHN (EudraCT No. 2016-004787-20). Several phase I and phase II RCTs have been conducted on neoadjuvant therapies with ICI in patients with HNSCC and all achieved promising results with response in up to 52% of cases (11–16). Induction with ICI is likely to be a more effective method of tumor control with fewer side effects compared to adjuvant immunotherapy, as more tumor antigens are present when the tumor is still *in situ* with its higher mass (4, 17, 18). It is expected that the immune system could be better protect against tumor recurrence in the future, whenever immunologic memory develops most early (19, 20). Indeed, immune evasion by the tumor is enhanced by overexpression of the immune checkpoint molecule programmed-death-ligand-1 (PD-L1) on tumor cells and/or tumor-infiltrating immune cells, which is found in around 55% of HNSCC patients and especially after prolonged presence of the tumor. Hence, a combination of immunotherapy with chemotherapy and/or radiotherapy is considered particularly promising in earlier (upfront or neoadjuvant) settings because immunogenic antigens are released during chemotherapy and radiotherapy, which putatively may enhance the effect of ICI (21), and the negative effect exerted by through chemo-induced PD-L1 expression on tumor cells can be abrogated by ICI (22–24). Binding of PD-L1 to its receptor, programmed-death-1 (PD-1) on T, B and NK cells, results in inhibition of proliferation and effector function of these cells (25–27). Thus, cancer may be able to escape immune-mediated destruction (28–30). Although, high PD-L1 expression on tumor and/or immune cells correlates with improved response to anti-PD-1 blockade (28, 31, 32), there is still an immense need for research to improve OS and QoL of patients. Even though therapies performed with an anti-PD-1-ICI in patients with advanced HNSCC have resulted in prolonged survival in the palliative setting compared to standard therapy (33), not all patients respond equally, and predicting response to PD-1 inhibitor therapy remains challenging (9, 34–38). PD-L1 is currently the only clinically available and routinely used biomarker for optimizing patient selection for anti-PD-1-ICI in non-small cell lung cancer (NSCLC) (39), gastric cancer and HNSCC (9, 34, 35, 38, 40, 41). As use of PD-L1 expression as biomarker is only able to enrich responders among treated patients, clinical characteristics such as T and N category still play the most important role in treatment decision for curative HNSCC (42–47), as (compared to other solid tumors) reliable biomarkers like micro-satellite instability (*e.g.* MSI) for treatment stratification are very infrequent or even missing; *ex-vivo* testing and monitoring of *in vivo* responses via liquid biopsies, however, may overcome the dilemma of missing biomarkers.

Indeed, diverse *ex-vivo* and *in-vitro* testing methods have already been used in the development of pharmaceuticals and have the potential to facilitate or even allow for better stratification of tumor therapies for particular HNSCC subgroups, especially for the use of immunomodulators such as ICI. Reliable prognostication prior to initiation of therapy in patients with cytostatics and targeted therapeutics is useful because HNSCC have a very heterogeneous biology and often low response rates to a given pharmaceutical. However, to date, *ex-vivo* assays have mainly not allowed reliable prediction of treatment success in a clinical context due to so far not available validation studies. Improved methods, however, are leading to a re-evaluation of the *ex-vivo* approach with expanded analysis of the antitumor immune response (48).

The randomized phase II LOP RCT DELOS-II investigated the effect of adding cetuximab to already well-performing therapy with TPF or TP and radiotherapy, also with the hope of LOP in locoregionally advanced LHSCC (2–6, 10, 10). *Ex-vivo* investigations of biopsy samples showed high positive predictive value of reduced colony formation for successful curative treatment and LOP in DeLOS-II (49). Both RCTs, KEYNOTE-048 and DELOS II, form the basis of the randomized controlled phase II LOP trial ELOS in advanced stage III, IVA/B LHNSCC resectable only by total laryngectomy having PD-L1 expression with CPS ≥ 1. ELOS investigates the effect of up to 17 cycles pembrolizumab added to TP treatment. However, as biomarkers allowing to identify responders or nonresponders are missing, we are searching for blood-derived biomarkers as well as *ex-vivo* chemoresponse testing to predict or at least monitor the response to ICI.

Cytokines may have a role as potential biomarkers (50–55) as they are overproduced by many solid tumors including HNSCC (56). Cytokine expression is associated with inflammation and angiogenesis involved in progression of cancer and along growth and progression of the cancer can increase from physiologic pg/ml levels to more than 1000-fold concentrations (57, 58). Our previous research on cytokine expression patterns *in vivo* and *ex vivo* already showed that response to PD-1 blockade is accompanied by shifts in cytokine concentrations closely linked to patient outcome, OS in particular (59). Röhl et al. demonstrated differences between non-responders and responders to PD-1 ICB in terms of levels of various pro-inflammatory and pro-angiogenic cytokines and growth factors before and during/after starting therapy, including MCP-1 (CCL-2) and VEGF-A, but also IFN-γ and chemokines such as IL-8 (CXCL-8) and IP-10 (CXCL-10). Such cytokine expression patterns allow to identify responders with improved survival compared to non-responders. Indeed, there was improved outcome in patients with a low IFN-γ concentration before and after ICB, and especially long-term OS after ICB, whenever serum or plasma concentrations of VEGF, IL-6 and IL-8 were rather low. We recently demonstrated that whenever PD-1-ICB failed to suppress MCP-1 levels, the outcome of HNSCC patients was impaired. Likewise, an increase in IL-6, IL-8 and VEGF was linked to impaired OS (63). In light of our previous research on PD-1 blockade on HNSCC *ex vivo* that revealed subgroups of patients with different response patterns in terms of cytokine release and colony formation *ex vivo* we speculated that response patterns observed in short-time *ex-vivo* tests could be prognostic for outcome independent of treatment.

The question of whether these groups can be identified after treatment *ex vivo* of their tumors remains open for particular treatments. Despite insignificant differences between patients treated with or without chemotherapy (59), a benefit of the combination of pembrolizumab and TP, as will be used in ELOS, remains to be demonstrated. In preparation of the ELOS trial and to check the feasibility of a reliable testing of response to per protocol treatment, we analyzed response of unselected HNSCC biopsies to pembrolizumab, TP and pembrolizumab plus TP to find out if an improved response to the combined treatment can be detected *ex vivo*.

## Materials and methods

### Study population and patient samples

The study was approved by the ethics committee of the University of Leipzig (vote NICEI-CIH 341-15-ff) and conducted according to the guidelines of the Declaration of Helsinki. Included in the study were samples from histopathological confirmed HNSCC treated in curative or palliative setting at Leipzig University Hospital. From January 2019 to September 2020, patients were informed and gave their written consent for the collection and examination of a tumor sample. Samples of 54 patients, among them 47 HNSCC patients were obtained from tissue biopsies token during panendoscopy or definitive surgery at the Otolaryngology or oral and maxillofacial surgery clinic of the University Hospital Leipzig. The patients were treated according to the decision in the multidisciplinary tumor board (MDTB); the MTDB was blinded regarding the outcome of *ex-vivo* tests (see below). The tumor database of the Department of Otolaryngology served as the source of all clinical data, including staging according to Union for International Cancer Control (UICC) criteria, TNM categories, and clinical follow-up data. The enrolled patients’ data were extracted from the tumor database and curated by JW & GW with contributions of LR and TW the patients’ characteristics at the time of registration for the study (at which also the biopsy was taken) is shown in **Table 1**.

**Table 1:** Distribution according to numbers (*n*) and percentage (%) as well as odds ratio (OR) and 95% confidence intervals (95% CI) and 2-sided *p*-value derived from chi-squared tests for categorical measures of various clinical and epidemiologic characteristics and outcome of head and neck squamous cell carcinoma patients providing biopsies for short-time *ex-vivo* chemoresponse testing according the FLAVINO protocol.

Covariate	Characteristic	Total *n* (%)	LHSCC *n* (%)	Other HNSCC *n* (%)	OR (95% CI)	*p v*alue
Sex						0.391
	Male	40 (85.1)	12 (92.3)	28 (82.4)	1 (95% CI 0.384 - 2.602)	
	Female	7 (14.9)	1 (7.7)	6 (17.6)	2.571 (95% CI 0.279 - 23.73)	
Age						0.484
	< 50	4 (8.5)	0 (--)	4 (11.8)	1 (95% CI 0.016 - 62.30)#	
	51 < 60	22 (46.8)	6 (46.2)	16 (47.1)	0.296 (95% CI 0.014 - 6.375)	
	61 < 70	16 (34)	6 (46.2)	10 (29.4)	0.185 (95% CI 0.008 - 4.079)	
	≥ 70	5 (10.6)	1 (7.7)	4 (11.8)	0.444 (95% CI 0.012 - 17.13)	
Smoking						0.811
	No	12 (25.5)	3 (23.1)	9 (26.5)	1 (95% CI 0.158 - 6.347)	
	Yes	35 (74.5)	10 (76.9)	25 (73.5)	0.833 (95% CI 0.186 - 3.729)	
Smoking						0.070
	Never	12 (25.5)	3 (23.1)	9 (26.5)	1 (95% CI 0.158 - 6.347)	
	Former	6 (12.8)	4 (30.8)	2 (5.9)	0.167 (95% CI 0.02 - 1.42)	
	Current	29 (61.7)	6 (46.2)	23 (67.6)	1.278 (95% CI 0.262 - 6.239)	
Pack years smoking history						0.813
	> 30 PY	23 (48.9)	6 (46.2)	17 (50)	1 (95% CI 0.268 - 3.729)	
	< 30 PY	24 (51.1)	7 (53.8)	17 (50)	0.857 (95% CI 0.238 - 3.086)	
Alcohol						0.931
	Never	14 (29.8)	4 (30.8)	10 (29.4)	1 (95% CI 0.194 - 5.154)	
	Former	6 (12.8)	2 (15.4)	4 (11.8)	0.8 (95% CI 0.102 - 6.25)	
	Current	27 (57.4)	7 (53.8)	20 (58.8)	1.143 (95% CI 0.27 - 4.843)	
Alcohol (g/day)						0.265
	Never	14 (29.8)	4 (30.8)	10 (29.4)	1 (95% CI 0.194 - 5.154)	
	1 - 30	11 (23.4)	2 (15.4)	9 (26.5)	1.8 (95% CI 0.263 - 12.29)	
	31 - 60	5 (10.6)	0 (--)	5 (14.7)	4.714 (95% CI 0.213 - 104.5)#	
	> 60	17 (36.2)	7 (53.8)	10 (29.4)	0.571 (95% CI 0.126 - 2.584)	
Smoking & alcohol consumption						0.824
	Neither risk factor	10 (21.3)	3 (23.1)	7 (20.6)	1 (95% CI 0.148 - 6.772)	
	One risk factor	25 (53.2)	6 (46.2)	19 (55.9)	1.357 (95% CI 0.265 - 6.958)	
	> 30 PY, > 60 g/day	12 (25.5)	4 (30.8)	8 (23.5)	0.857 (95% CI 0.141 - 5.229)	
T category (8^th^ ed.)						0.306
	T1	4 (8.5)	2 (15.4)	2 (5.9)	1 (95% CI 0.063 - 15.98)	
	T2	14 (29.8)	4 (30.8)	10 (29.4)	2.5 (95% CI 0.256 - 24.37)	
	T3	12 (25.5)	1 (7.7)	11 (32.4)	11 (95% CI 0.646 - 187.1)	
	T4	7 (14.9)	2 (15.4)	5 (14.7)	2.5 (95% CI 0.194 - 32.19)	
	T4a	8 (17)	4 (30.8)	4 (11.8)	1 (95% CI 0.091 - 11.02)	
	T4b	2 (4.3)	0 (--)	2 (5.9)	5 (95% CI 0.15 - 166.5)#	
T1 - T3 vs. T4						0.378
	T1 - T3	30 (63.8)	7 (53.8)	23 (67.6)	1.792 (95% CI 0.486 - 6.615)	
	T4	17 (36.2)	6 (46.2)	11 (32.4)	1 (95% CI 0.245 - 4.083)	
N category (8^th^ ed.)						0.477
	N0	16 (34)	6 (46.2)	10 (29.4)	1 (95% CI 0.239 - 4.184)	
	N1	14 (29.8)	4 (30.8)	10 (29.4)	1.5 (95% CI 0.322 - 6.991)	
	N2	1 (2.1)	0,5 (0)	1,5 (2.9)	1.857 (95% CI 0.065 - 52.76)	
	N2b	1 (2.1)	1,5 (7.7)	0 (--)	0.206 (95% CI 0.007 - 5.86)#	
	N2c	5 (10.6)	1 (7.7)	4 (11.8)	2.4 (95% CI 0.215 - 26.82)	
	N3a	1 (2.1)	0 (--)	1 (2.9)	1.857 (95% CI 0.065 - 52.76)#	
	N3b	9 (900)	1 (7.7)	8 (23.5)	4.8 (95% CI 0.475 - 48.46)	
N3						0.159
	other	37 (78.7)	12 (92.3)	25 (73.5)	0.231 (95% CI 0.026 - 2.043)	
	N3	10 (21.3)	1 (7.7)	9 (26.5)	1 (95% CI 0.054 - 18.57)	
M						0.820
	M0	44 (93.6)	12 (92.3)	32 (94.1)	1.333 (95% CI 0.11 - 16.09)	
	M1	3 (6.4)	1 (7.7)	2 (5.9)	1 (95% CI 0.034 - 29.80)	
UICC stage 8^th^ ed.						0.726
	UICC I	7 (14.9)	2 (15.4)	5 (14.7)	1 (95% CI 0.098 - 10.16)	
	UICC II	4 (8.5)	1 (7.7)	3 (8.8)	1.2 (95% CI 0.073 - 19.63)	
	UICC III	16 (34.0)	6 (46.2)	10 (29.4)	0.667 (95% CI 0.097 - 4.58)	
	UICC IVA	6 (12.8)	2 (15.4)	4 (11.8)	0.8 (95% CI 0.076 - 8.474)	
	UICC IVB	11 (23.4)	1 (7.7)	10 (29.4)	4 (95% CI 0.288 - 55.47)	
	UICC IVC	3 (6.4)	1 (7.7)	2 (5.9)	0.8 (95% CI 0.044 - 14.64)	
IVB						0.182
	other	33 (70.2)	11 (84.6)	22 (64.7)	0.333 (95% CI 0.063 - 1.758)	
	IVB or IVC	14 (29.8)	2 (15.4)	12 (35.3)	1 (95% CI 0.12 - 8.307)	
Grading						0.579
	G1	2 (4.3)	1 (7.7)	1 (2.9)	1 (95% CI 0.02 - 50.4)	
	G2	23 (48.9)	5 (38.5)	18 (52.9)	3.6 (95% CI 0.19 - 68.34)	
	G3	22 (46.8)	7 (53.8)	15 (44.1)	2.143 (95% CI 0.116 - 39.47)	
Lymphatic infiltration						0.534
	L1	33 (70.2)	10 (76.9)	23 (67.6)	1 (95% CI 0.35 - 2.857)	
	L0	14 (29.8)	3 (23.1)	11 (32.4)	1.594 (95% CI 0.364 - 6.981)	
p16 IHC						0.324
	p16+	12 (25.5)	2 (15.4)	10 (29.4)	1 (95% CI 0.117 - 8.56)	
	p16 neg (or unknown)	35 (74.5)	11 (84.6)	24 (70.6)	0.436 (95% CI 0.082 - 2.336)	
PD-L1 IHC						0.493
	CPS ≥ 1	18 (38.3)	6 (46.2)	12 (35.3)	1 (95% CI 0.25 - 3.999)	
	CPS < 1	29 (61.7)	7 (53.8)	22 (64.7)	1.571 (95% CI 0.429 - 5.752)	
Extranodal extension (ENE)						0.179
	No ENE	20 (42.6)	6 (46.2)	14 (41.2)	1 (95% CI 0.259 - 3.867)	
	ENE+	12 (25.5)	1 (7.7)	11 (32.4)	4.714 (95% CI 0.492 - 45.15)	
	N0 (no ENE)	15 (31.9)	6 (46.2)	9 (26.5)	0.643 (95% CI 0.157 - 2.627)	
Resection margins						0.207
	R0	37 (78.7)	12 (92.3)	25 (73.5)	1 (95% CI 0.378 - 2.647)	
	R1	3 (6.4)	1 (7.7)	2 (5.9)	0.96 (95% CI 0.079 - 11.66)	
	No surgery	7 (14.9)	0 (--)	7 (20.6)	7.353 (95% CI 0.388 - 139.3)#	
Op						0.217
	Yes	38 (80.9)	12 (92.3)	26 (76.5)	1 (95% CI 0.38 - 2.631)	
	No	9 (19.1)	1 (7.7)	8 (23.5)	3.692 (95% CI 0.414 - 32.94)	
Anti-PD-1						0.514
	Yes	5 (10.6)	2 (15.4)	3 (8.8)	1 (95% CI 0.08 - 12.55)	
	No	42 (89.4)	11 (84.6)	31 (91.2)	1.879 (95% CI 0.276 - 12.77)	
Cisplatin						0.596
	Yes	21 (44.7)	5 (38.5)	16 (47.1)	1 (95% CI 0.242 - 4.138)	
	No	26 (55.3)	8 (61.5)	18 (52.9)	0.703 (95% CI 0.191 - 2.592)	
RT						0.917
	Yes	32 (68.1)	9 (69.2)	23 (67.6)	1 (95% CI 0.336 - 2.974)	
	No	15 (31.9)	4 (30.8)	11 (32.4)	1.076 (95% CI 0.271 - 4.276)	
Overall survival (OS)						0.628
	Alive	23 (48.9)	5 (38.5)	18 (52.9)	1 (95% CI 0.246 - 4.06)	
	NCRD	5 (10.6)	2 (15.4)	3 (8.8)	0.417 (95% CI 0.054 - 3.221)	
	CRD	19 (40.4)	6 (46.2)	13 (38.2)	0.602 (95% CI 0.151 - 2.404)	
PFS						0.677
	PFS event	23 (48.9)	7 (53.8)	16 (47.1)	1 (95% CI 0.285 - 3.512)	
	No event	24 (51.1)	6 (46.2)	18 (52.9)	1.313 (95% CI 0.364 - 4.728)	
LRFS (LC)						0.900
	PFS event	21 (44.7)	6 (46.2)	15 (44.1)	1 (95% CI 0.262 - 3.815)	
	No event	26 (55.3)	7 (53.8)	19 (55.9)	1.086 (95% CI 0.301 - 3.919)	
LRRFS (LRC)						0.900
	PFS event	21 (44.7)	6 (46.2)	15 (44.1)	1 (95% CI 0.262 - 3.815)	
	No event	26 (55.3)	7 (53.8)	19 (55.9)	1.086 (95% CI 0.301 - 3.919)	
NRFS (NC)						0.421
	PFS event	14 (29.8)	5 (38.5)	9 (26.5)	1 (95% CI 0.213 - 4.693)	
	No event	33 (70.2)	8 (61.5)	25 (73.5)	1.736 (95% CI 0.449 - 6.713)	
DMFS						0.075
	PFS event	10 (21.3)	5 (38.5)	5 (14.7)	1 (95% CI 0.173 - 5.772)	
	No event	37 (78.7)	8 (61.5)	29 (85.3)	3.625 (95% CI 0.837 - 15.70)	

### Materials

#### FLAVINO assay

The FLAVINO assay is a short-time *ex-vivo* assay to test HNSCC regarding response to various treatments. To this end, colony formation and cytokine release of tumor cells exposed to various therapeutic agents are compared with their controls. This allows also the estimation of combinatory effects. Immediately after excision of the biopsy during panendoscopy or tumor surgery, the viable samples were put into cell culture medium and transferred at room temperature into the lab. The cell culture medium was a custom-made phenol- and riboflavin-free RPMI1640 (Bio & Sell GmbH, Feucht, Germany) containing 10% fetal calf serum (FCS; Anprotec, Bruckberg, Germany) with streptomycin, penicillin, amikacin, and nystatin C (all Sigma-Aldrich Chemie GmbH, Deisenhofen, Germany). All steps in handling the biopsy and the cells obtained from the tumor sample were executed under flavin-protective conditions (illumination only by sodium discharge lamps emitting monochromatic light at a wavelength of λ = 589 nm; Philips Medical Systems DMC GmbH, Hamburg, Germany). Mechanically disintegrated tumor tissue was digested via overnight incubation with 230 mIU/ml collagenase IV (Sigma-Aldrich Chemie GmbH, Deisenhofen, Germany) before cell counting utilizing 1:10 diluted Guava^®^ ViaCount™ reagent (Luminex Corp., Austin, TX) for counting of viable cells in the Guava easyCyte 5HT flow-cytometer (Luminex). Flat-bottom cell culture-plates (TPP, Trasadingen, Switzerland) were pre-coated with laminin, collagen I and fibronectin (all from Roche, Mannheim, Germany). Pre-diluted pharmaceuticals were pipetted into six cavities each before seeding of 3x10^4^ viable cells per well to adjust to final concentrations of either 50 µg/ml pembrolizumab (Pemb), the (binary) TP combination of docetaxel (275 nM) and cisplatin (3333 nM) or the ternary combination of 50 mg/ml pembrolizumab plus TP at the same concentrations or medium only (control for reference) each in six replicates. After a further three days of incubation under standard conditions (36.5°C, humidified atmosphere, 3.5% CO_2_), 200 µl of culture supernatants were collected and transferred to 384-well plates and stored frozen at -80°C for subsequent cytokine measurement by indirect sandwich enzyme-linked immunosorbent assay (ELISA, see below). Furthermore, cells were step-wise fixed with ethanol (40%, 70%, and 96% ethanol), and air-dried. Before subsequent colony counting, wells were blocked with an assay buffer containing 1% FCS (v/v) to prevent unspecific binding of anti-cytokeratin (Santa-Cruz Biotechnology, Inc., Santa-Cruz, USA) and FITC-labeled secondary antibody (Thermo Fisher Scientific GmbH, Dreieich, Germany). The antibodies were each diluted 1:800 in phosphate-buffered saline (PBS) containing 0.5% FCS and 0.05% Tween-20™. Stained epithelial cells were counted using a fluorescence microscope (Axiovert, Carl Zeiss Microscopy Deutschland GmbH, Oberkochen, Germany).

#### ELISA

Indirect sandwich ELISAs were performed to measure cytokine concentrations in cell-free supernatants of cell cultures harvested 72 h after exposure to drugs and drug combinations. Using OptEIA™ kits (BD GmbH, Heidelberg, Germany) IL-6, IL-8, IFN-γ, IP-10, MCP-1, and TNF-α, and VEGF-EDK kits for VEGF_165_ (#900-K10, PeproTech GmbH, Hamburg, Germany), cytokine concentrations were measured according to the manufacturer’s instructions but using tetramethyl benzidine (TMB 1-Step™ Ultra, Thermo Fisher Scientific) as substrate. Furthermore, measurements were performed at optical densities of λ_1_ = 450 nm and λ_2_ = 620 nm using the Synergy2™ multimode microplate reader (BioTek Instruments, Inc., Winooski, VT, USA). We used 4-parameter calibration curves to calculate pg/ml concentrations using Gen5™ software (BioTek Instruments, Inc., Winooski, VT, USA). The lower limit of detection (LLD) and the lower limit of quantification (LLQ) for cytokine detection was always < 4 pg/ml.

### Evaluation of drug combinations and statistical analysis

For the objective assessment of the interactions between the drugs used, the changes from baseline (untreated control) were used to obtain delta values, which were used to calculate the interaction measure *q* (60–67) using the following formula:


(1) q= P(A+B)/((P(A) + P(B) − P(A) x P(B))

wherein P(A) represents the effect of compound A (for instance, pembrolizumab), P(B) the effect of compound B (here: TP), and P(A+B) the effect of A and B in mixture with the same concentrations (pembrolizumab + TP). The evaluation of the respective mode of action considers the uncertainty of the measurements with regard to the interpretation of the value for *q*: a result of the equation of *q* < 0.85 results in antagonism of the effect ratios, while *q* = 0.85 to 1.15 reflects additivity, and *q* > 1.15 indicates synergism. Cut-off analyses were performed for all experiments of sufficient colony formation (CFec ≥ 4). A summary of results for the individual cytokines is shown in graphs depicting the calculation of mean, standard deviation and confidence interval but also median and interquartile range (IQR). Colony formation values were analyzed with a two-tailed *t*-test for paired samples (SPSS Statistics 29.0 for Windows, SPSS Inc., Chicago, IL, USA). When *p* was < 0.05, the results were considered significant. Patient characteristics and follow-up data were analyzed in relation to the results from ELISA measurements and categorization according to receiver-operating characteristic (ROC) curves as described above. We also analyzed clinical characteristics of patients, and lifestyle-associated risk factors (daily alcohol consumption categorized in 0, 1-30 g, 31-60 g, > 60 g) and status (never, former, current), tobacco smoking (total number of pack years smoked during lifetime), smoking status (never, former, current smoker). Clinical characteristics of patients included age; sex; T, N and M categories; HPV status (according to p16 immune histochemistry), and treatment modalities (curative vs. palliative setting). Associations between categorical variables were examined by *Pearson’s Chi-square* test. We calculated OS as time from date of biopsy to date of death (event), or end of follow-up (censored); DSS as time from date of biopsy to date of cancer-related death (event) censoring other causes of death or end of follow-up; PFS from date of biopsy to date of relapse or progressing disease or death from any cause (event), or end of follow-up (censored). Local relapse-free survival (LRFS) was calculated from date of biopsy to date of local relapse (within 2 cm resection margins) or progressing disease or death from any cause (event), or end of follow-up (censored). Nodal relapse-free survival (NRFS) was calculated from date of biopsy to date of diagnosis of locoregional relapse (local metastasis in locoregionary lymph nodes, independent of ipsilateral or contralateral) or progressing disease or death from any cause (event), or end of follow-up (censored). Loco-regional relapse-free survival (LRRFS) was calculated from date of biopsy to date of LRFS or NRFS, whatever came first, or progressing disease or death from any cause (event), or end of follow-up (censored). Distant metastasis-free survival (DMFS) was calculated from date of biopsy to date of diagnosis of distant metastasis (M1, event) or death from any cause (event), or end of follow-up (censored).

We analyzed survival using Kaplan-Meier cumulative survival plots applying log-rank tests and hazard ratios (HR) using multivariate Cox proportional hazard regression models (76) utilizing the conditional logistic regression step-wise forward method, and bootstrapping for internal validation (SPSS version 29, IBM Corporation, Armonk, New York). We considered *p* < 0.05 from 2-sided tests as significant.

## Results

A total of *n* = 47 HNSCC patients (40 males, 7 females) out of *N* = 54 samples (87.0%) obtained allowed for cytokine measurements, whereas only 12 (22.2%) were also reliably analyzable regarding colony formation of adherent epithelial cells (CFec) with mean CFec ≥ 4/well in the 6 replicate wells of sham-treated controls (**Supplementary Figure S1**). As the small number of 12 HNSCC samples did not allow for suitable subgroup analyses, we focused on treatment effects on cytokine production and compared 13 LHSCC and 34 HNSCC emerging from other sites. The clinical and epidemiological characteristics including distribution of lifestyle-related risk factors, TNM categories and stage as well as treatment of both groups were comparable with unadjusted *p* ≥ 0.159 (**Table 1**). The outcome was also comparable with DMFS being the only measure showing a trend to impaired outcome in LHSCC (*p* = 0.075).

**Figure 1:**
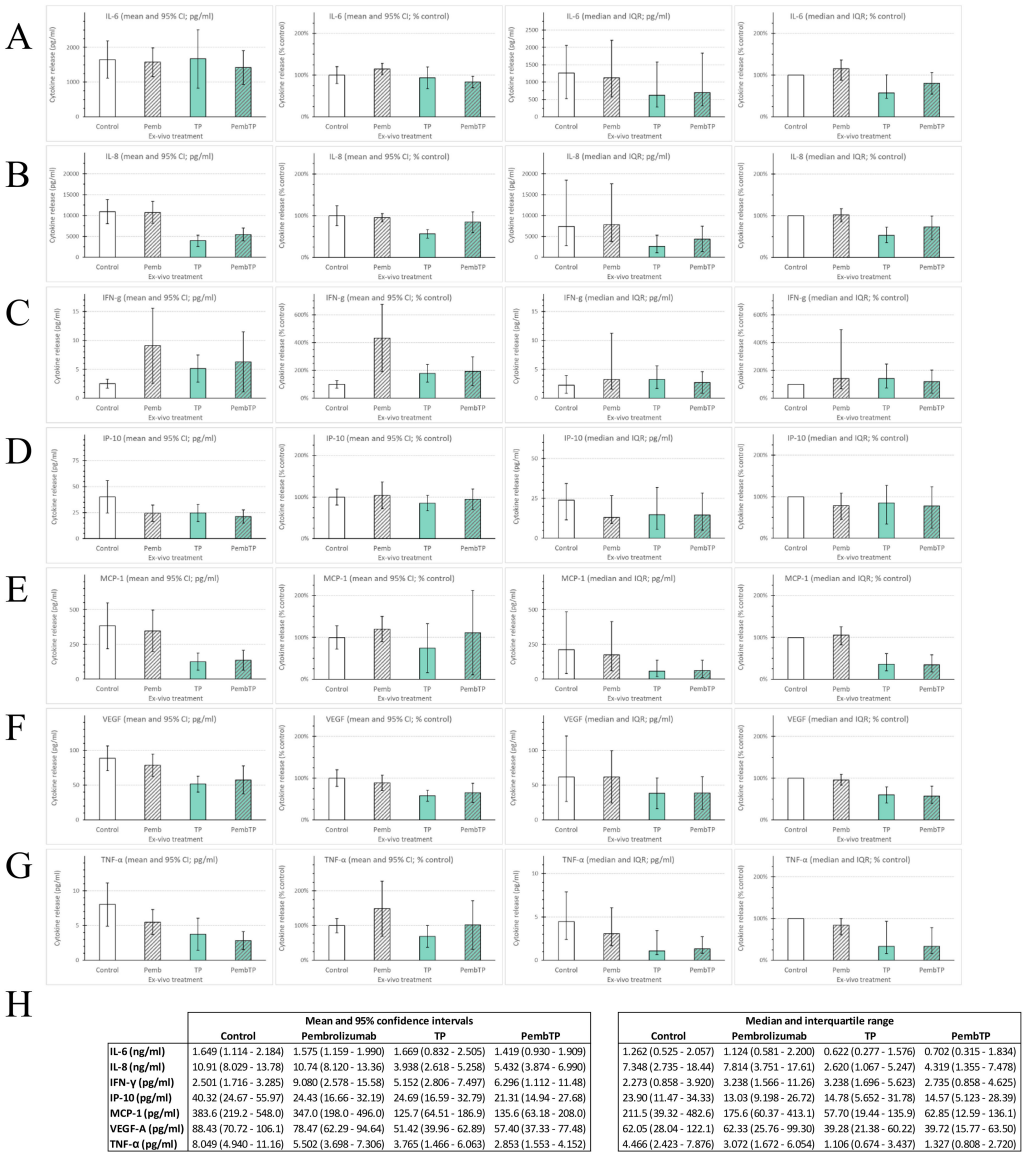
Cytokine production of head and neck squamous cell carcinoma of 47 patients treated *ex vivo* according the FLAVINO protocol with immune-checkpoint blockade (ICB) utilizing the anti-PD-1 antibody pembrolizumab either alone (Pemb; 50 ng/ml pembrolizumab mono), TP or PembTP, Pemb combined with half maximum tolerable plasma concentrations of docetaxel (T; 275 nM) and cisplatin (P; 3.33 µM). Mean concentrations (pg/ml) and normalized mean (% of untreated control) as well as median concentrations (pg/ml) and normalized mean (% of untreated control) inclusive 95% CI and interquartile range (IQR) measured in 72-hours supernatants are shown for **(A)** interleukin 6 (IL-6); **(B)** IL-8; **(C)** interferon gamma (IFN-γ); **(D)** interferon-induced protein 10 (IP-10; CXCL10); **(E)** monocyte chemo-attractant protein 1(MCP-1; CCL2); **(F)** vascular endothelial growth factor A (VEGF); **(G)** tumor-necrosis-factor alpha (TNF-α); **(H)** mean and 95% CI (left) and median and IQR (right).

**Figure 1** summarizes the treatment-related differences in cytokine production of the 47 samples based on mean and 95% confidence intervals (95%-CI) but also median and interquartile range (IQR) for the seven cytokines measured.

Treatment with pembrolizumab alone had no generalizable effect on production of IL-6, IL-8, MCP-1, TNF-α and VEGF, while IFN-γ was strongly induced. Due to unexpectedly high concentrations of IP-10 produced by sham-treated (medium only) controls, only few samples demonstrated increased IP-10 production without substantial impact toward a generally enhanced IP-10 production according to higher 95%-CI or IQR. The binary combination of docetaxel and cisplatin (TP) demonstrated strong heterogeneity of samples with respect to production of IL-6, as some samples responded with strong induction of IL-6 leading to nearly unchanged mean (1.669 vs. 1.649 ng/ml) but widened 95%-CI (0.832-2.505 vs. 1.114-2.184 ng/ml in controls). The median of 0.622 vs. 1.262 ng/ml was halved (**Figure 1H**). With IFN-γ again representing the only exception, the production of the other cytokines was found to be suppressed. However, comparing mean and median revealed heterogeneity also in this regard. The stimulating effect of TP on IFN-γ production was below that observed in pembrolizumab-treated samples. However, the combination of TP and pembrolizumab resulted in even stronger deviating amounts of IFN-γ production measured after 72 hours. Whereas production of IL-8, TNF-α and VEGF demonstrated antagonism, as the strong suppression through TP was mostly reduced by simultaneously present pembrolizumab (**Figures 1 B, D, F–H**), IP-10 release was only slightly modified (often within the range of measurement uncertainty). The production of MCP-1 and IL-6, however, demonstrated also deviating interaction of TP and pembrolizumab when comparing individual samples (compare **Supplementary Table S1**, available online). Therefore, we systematically investigated differences in outcome of patients related to *ex-vivo* response-patterns of their tumors. To this end, we used receiver-operating-characteristic (ROC) curves for a binary split of samples according to optimum cut-offs. This, however, failed to demonstrate significant outcome differences (with all *p* ≥ 0.2).

**Figure 2:**
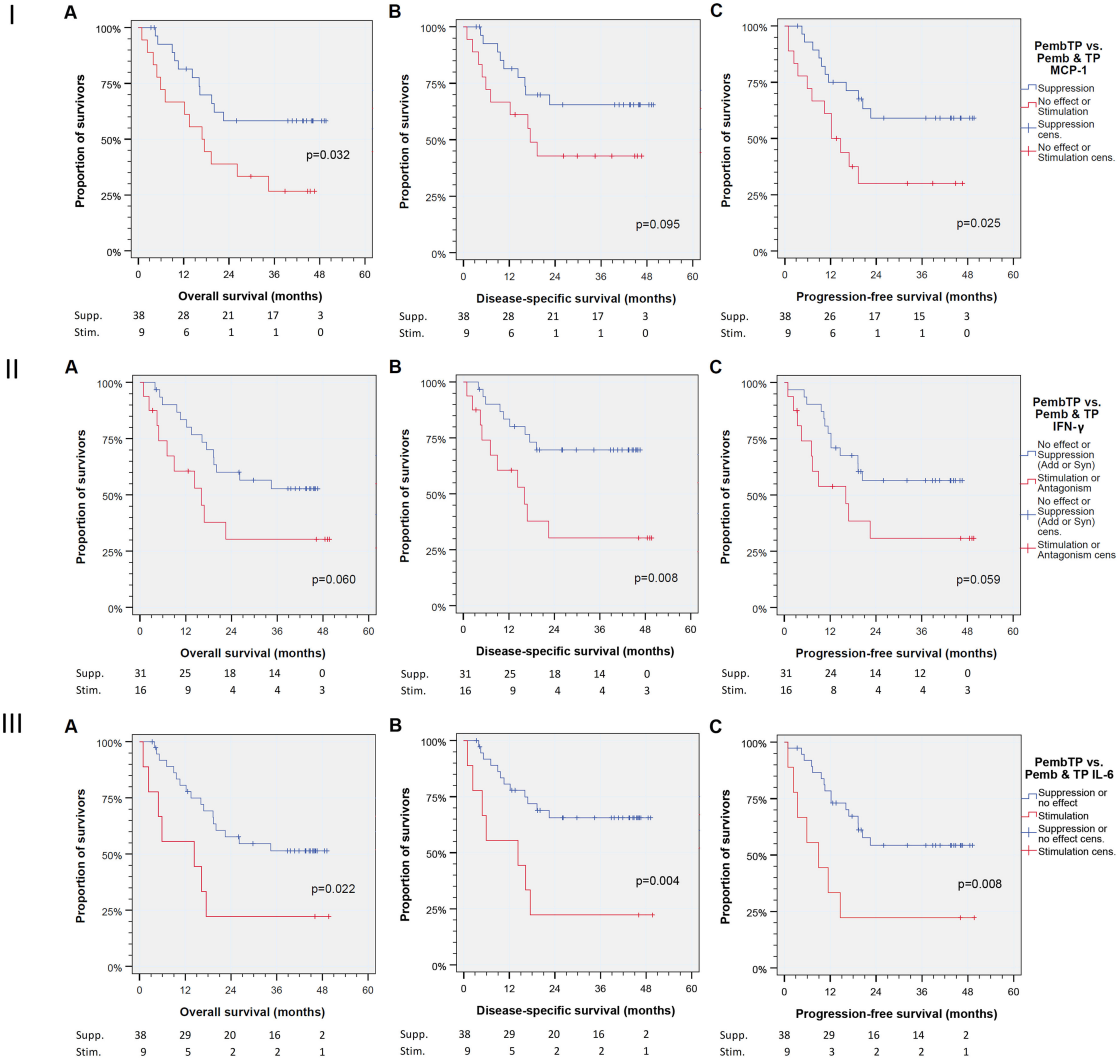
Kaplan-Meier cumulative survival plots for (from left to right) overall, disease-specific, and progression-free survival of 47 patients treated *ex vivo* with immune-checkpoint inhibitor (ICI) utilizing the anti-PD-1 antibody pembrolizumab either alone (pembrolizumab mono) vs. TP or pembrolizumab combined with docetaxel (T) and cisplatin (P) according to half maximum tolerable plasma concentration (275 nM and 3.33 µM, respectively) were compared. Binary split of the cohort was according to the mode of action, suppression (Supp.) of MCP-1 or no effect or suppression of IFN-γ or IL-6 production by pembrolizumab + TP vs. those with stimulation (Stim.). Numbers for patients at risk are provided for **(I)** monocyte chemoattractant protein 1 (MCP-1; CCL2); **(II)** interferon gamma (IFN-γ); **(III)** interleukin 6 (IL-6). *P* values shown are from log rank tests (2-sided).

**Figure 3:**
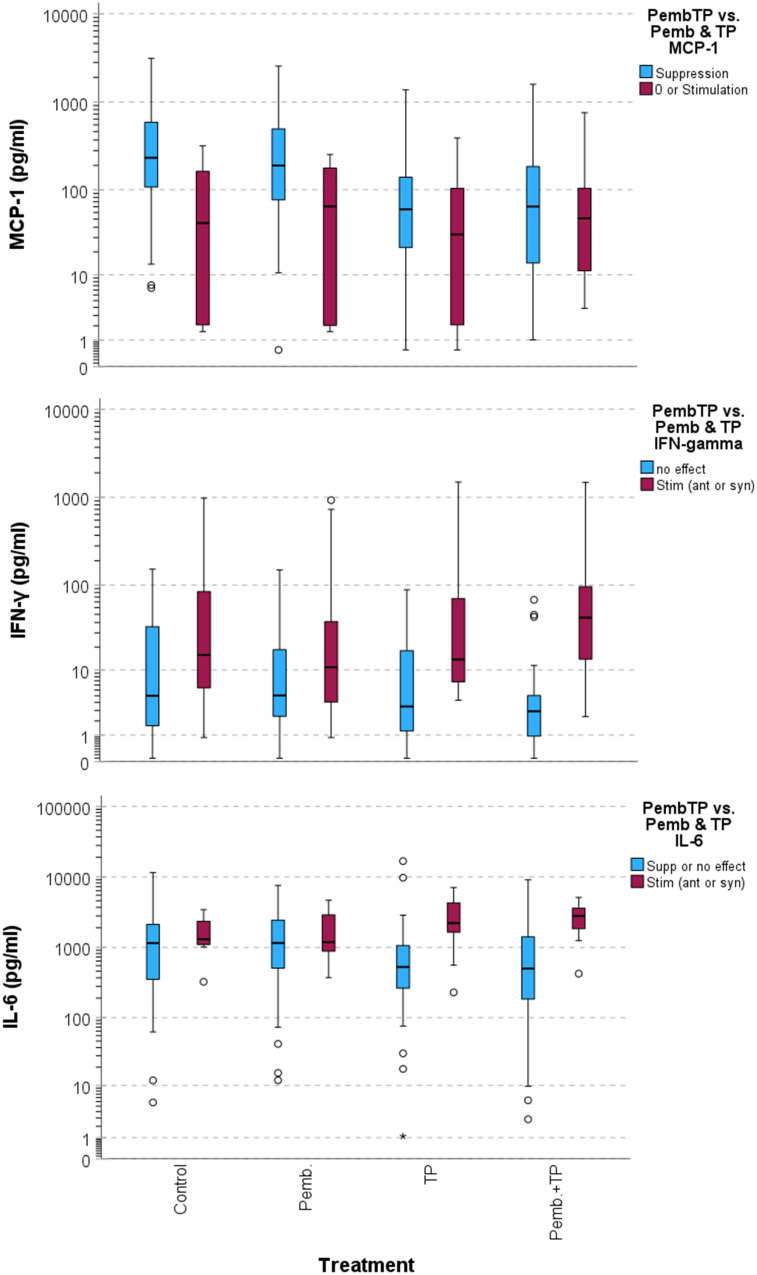
Boxplots demonstrating deviating cytokine production of head and neck squamous cell carcinoma in treatment response groups according to binary classification in **Figure 2**.

In sharp contrast, the response to TP plus pembrolizumab vs. TP and pembrolizumab alone as reflected by changes in production of MCP-1, IFN-γ and IL-6 was related to deviating outcome with significant differences in OS, DSS and PFS (**Figure 2**). **Figure 3** shows boxplots for MCP-1, IFN-γ and IL-6 in response groups with deviating outcome. Obviously, patients with superior outcome (depicted blue) had rather high production of MCP-1 that was not substantially elevated by pembrolizumab but suppressed in response to TP, rather lower IL-6 and/or IFN-γ production that was suppressed by TP and/or pembrolizumab plus TP. Contrarily, patients with low MCP-1 production but elevated IL-6 and/or IFN-γ production (depicted red) were at risk for impaired outcome.

As, however, the response to TP plus pembrolizumab regarding MCP-1, IFN-γ and IL-6 production was not strongly correlated on the patient-individual level, we used Cox proportional hazard regression modeling to find out if response patterns emerging after combined treatment are independent predictors of outcome. By including all known clinical prognostic factors for OS of HNSCC patients, we found age at diagnosis, smoking history (according to number of pack-years tobacco smoking), daily alcohol consumption, T and N categories and p16-positivity but not localization in larynx/hypopharynx *vs.* other sites as well as the treatment setting (curative vs. palliative intend) were of prognostic relevance and represented independent predictors (*Pi*) for particular outcome measures, OS, DSS or PFS. The response-characteristics to TP plus pembrolizumab was introduced into modeling as binary categorized covariates (**Table 2**).

**Table 2:** Independent predictors (Pi) of overall survival (OS), disease-specific survival (DSS), and progression-free survival (PFS) of head and neck squamous cell carcinoma patients identified using multivariate Cox proportional hazard regression models build using the step-wise forward likelihood ratio method in SPSS v.29.

	Covariate	Ref.	Characteristic	*n*	Events *n* (%)	*P* value^#^	Cox univariate HR (95% CI)	*P* value^†^	Cox multivariate HR (95% CI)	*P* value^††^	Loss^‡^ in *χ* ^2^	*P* value^‡^	*P* value^‡‡^
**OS**	Age at diagnosis	Per year				--	1.009 (0.965 – 1.055)	0.6923	1.075 (1.018 - 1.135)	**0.0097**	6.751	**0.0090**	**0.0110**
	Pack years	Per year				--	1.024 (1.009 - 1.040)	**0.00142**	1.028 (1.010 - 1.046)	**0.0019**	10.469	**0.0010**	**0.0020**
	Treatment setting	curative	palliative	11	9 (81.8%)	**0.0203**	2.601 (1.126 - 6.007)	**0.02524**	3.444 (1.285 - 9.230)	**0.0139**	5.729	**0.0170**	**0.0250**
	p16 IHC	p16 negative or unknown	p16 positive	12	4 (33.3%)	0.2428	0.533 (0.182 - 1.56)	0.2505	0.223 (0.056 - 0.889)	**0.0335**	5.885	**0.0150**	0.0659
	MCP-1 PembTP vs. Pemb & TP	suppression	no effect or stimulation	9	8 (88.9%)	**0.0324**	3.337 (1.392 - 8.000)	**0.00691**	2.732 (1.031 - 7.240)	**0.0433**	3.673	0.0550	**0.0310**
	IFN-γ PembTP vs. Pemb & TP	no effect or suppression (add or syn)	stimulation or antagonism	16	10 (62.5%)	0.0596	2.153 (0.951 - 4.874)	0.06575	2.391 (1.005 - 5.689)	**0.0488**	3.709	0.0540	0.0849
	PembTP vs. Pemb & TP IL-6	no effect or suppression	stimulation (ant or syn)	9	7 (77.8%)	**0.0217**	2.714 (1.117 - 6.594)	**0.02746**	6.007 (2.024 - 17.83)	**0.0012**	9.327	**0.0020**	**0.0020**
**DSS**	Alcohol (g/day)	<30	≥30	22	13 (59.1%)	**0.0199**	2.989 (1.135 - 7.871)	**0.02668**	5.325 (1.753 - 16.18)	**0.0032**	10.038	**0.0020**	**0.0020**
	N category	N0	N+	31	16 (51.6%)	**0.0157**	4.091 (1.187 - 14.10)	**0.02565**	0.363 (0.184 - 0.717)	**0.0035**	11.771	**0.0010**	**0.0070**
	MCP-1 PembTP vs. Pemb & TP	suppression	no effect or stimulation	9	7 (77.8%)	**0.0095**	3.283 (1.276 - 8.448)	**0.0137**	6.392 (2.144 - 19.06)	**0.0009**	10.086	**0.0010**	**0.0010**
	IFN-γ PembTP vs. Pemb & TP	no effect or suppression (add or syn)	stimulation or antagonism	16	10 (62.5%)	**0.0082**	3.188 (1.288 - 7.890)	**0.01218**	2.592 (1.019 - 6.591)	**0.0455**	3.899	**0.0480**	0.0589
**PFS**	Alcohol (g/day)	< 30	≥ 30	22	14 (63.6%)	**0.0312**	2.447 (1.056 - 5.671)	**0.03696**	4.273 (1.675 - 10.90)	**0.0024**	9.868	**0.0020**	**0.0040**
	N category	N0	N+	31	19 (61.3%)	**0.0086**	3.872 (1.309 - 11.46)	**0.01445**	7.947 (2.319 - 27.24)	**0.0010**	14.748	**< 0.0001**	**0.0030**
	MCP-1 PembTP	suppression	no effect or stimulation	9	7 (77.8%)	**0.0253**	2.558 (1.036 - 6.319)	**0.04175**	4.698 (1.702 - 12.97)	**0.0028**	7.852	**0.0050**	**0.0010**

P values shown highlighted bold are significant with p < 0.05 in 2-sided statistics.

While some of the clinical *Pi* lost significance and were not included in the step-wise forward build Cox model, MCP-1 emerged as the only *Pi* of PFS (HR 4.698, 95%-CI 1.702-12.97; *p* = 0.0028), it was also *Pi* for DSS (HR 6.392, 95%-CI 2.144-19.06; *p* = 0.0009) and OS (HR 2.732, 95%-CI 1.031-7.240; *p* = 0.0433). Whereas IL-6 was *Pi* only for OS (HR 6.0077, 95%-CI 2.024-17.83; *p* = 0.0012), IFN-γ was *Pi* of DSS (HR 2.592, 95%-CI 1.019-6.591; *p* = 0.0455) and OS (HR 2.391, 95%-CI 1.005-5.689; *p* = 0.0488). Internal validation of the multivariate Cox proportional hazard regression models through bootstrapping applying 1,000 iterations revealed stability of the models and MCP-1 and IL-6 as *Pi* (all *p* ≤ 0.0310), while IFN-γ slightly missed this criterion with *p* = 0.0589 for DSS, and *p* = 0.0849 for OS (**Table 2**).

## Discussion

*Ex-vivo* response-evaluation of HNSCC utilizing ELISA for cytokine measurements allowed for analyzing deviating response to treatment with pembrolizumab, TP and pembrolizumab + TP. Suppression of MCP-1, IL-6 and IFN-γ production by pembrolizumab + TP exceeding the effect of TP or pembrolizumab were found to be independent predictors of outcome according to OS, DSS, and PFS since biopsy. The FLAVINO assay identified a fraction of HNSCC patients among those analyzed with impaired outcome independent from other clinical characteristics and treatment applied. This minor group of patients responded not the same way as the majority of HNSCC samples analyzed as they did not show suppression of MCP-1, IFN-γ, and IL-6 production by pembrolizumab + TP. We interpret this finding as patient-individual characteristic regarding deviating immune regulation. Reasons behind this finding could be manifold. It is well investigated that, after prolonged exposure, immune cells, CD4+ T-helper cells (Th) and cytotoxic T cells (Tc) become exhausted or may acquire resistance due to sustained signaling via their interferon receptors, IFNAR and IFNGR (68). We recently published findings about the prognostic value of IFN-γ measured before and during ongoing treatment with antibodies to PD-1, pembrolizumab or nivolumab, and impaired survival of patients with increased IFN-γ concentrations measured in EDTA-anticoagulated plasma (59). Within the same *in-vivo* study, suppression of MCP-1 concentrations ≥ 15% from baseline level was linked to improved survival. Within this *ex-vivo* study, we confirm that compared to pembrolizumab and TP reduced MCP-1 concentrations through combined pembrolizumab + TP is an indicator for HNSCC patients with rather good outcome according to OS, DSS, and PFS. IL-6 is a senescence marker also involved in inflammation and increases IFNGR signaling via facilitation of signal transducer and activator of transcription (STAT 1) phosphorylation (69). Hence, responding to pembrolizumab + TP with reduced production of MCP-1, IFN-γ, and IL-6 might reflect the presence of a well-functioning intratumoral immune infiltrate able to attack the cancerous epithelial cells in HNSCC. An important marker addressed in this study is the pro-inflammatory cytokine MCP-1, which is produced at the site of inflammation. MCP-1 binds to CCR2 and is involved in chemotactic recruitment of monocytes, macrophages and natural killer cells (70). In higher concentrations, this CC chemokine has an effect on the tumor environment and has demonstrated a correlation with tumor invasiveness, tumor angiogenesis and progression of the disease, and spread of metastases (71, 72). Interestingly, both tumor-supporting and tumor-inhibiting effects can be seen through the effects on different cell types and depending on the concentration. Significantly lower OS and DSS were observed in various studies when MCP-1 levels were elevated, suggesting that MCP-1 may be a good prognostic marker for HNSCC (73–75). Results have shown that chemokines produced by tumor cells promote the infiltration of immune cells into the tumor microenvironment (TME), and that MCP-1 (CCL2) plays a decisive role in this context (76). CCL-2 receptor (CCR2) expressing monocytes are recruited along a CCL2 gradient to the tumor periphery (77, 78) where they mature further in the TME and develop pro-tumoral functions (79, 80). This occurs through maturation into tumor-associated macrophages (TAMs), which further fosters tumor growth (79, 81, 82). Based on preclinical models, this growth could be impaired by blocking CCR2/CCL2 binding (83). A correlation between the concentration of CCL2, monocytes in the TME, and the suppression of the T-cell response can be observed in various cancer models (84–87). The resulting immunosuppressive mechanisms cause tumor progression. The CCR2-positive monocytes are thus an antagonist of the antigen-specific T cells.

IFN-γ is a key regulator centrally involved in the initiation of an antitumoral immune response but it can also exert pro-tumoral functions (88). It was shown that exposure to elevated IFN-γ levels and especially a prolonged exposure exerts selective immune pressure on the tumor cell leading to reduced expression of genes involved in antigen presentation, such as MHC class I (89). Persistent IFN-γ signaling also allows the tumor to acquire signal transducer and activator of transcription 1 (STAT1)-related epigenomic changes and augments expression of interferon-stimulated genes and ligands for multiple T-cell inhibitory receptors, which can be seen as a mechanism of adaptive resistance to checkpoint inhibitor therapy. Biomarkers for interferon-driven resistance are reported as being associated with clinical progression after anti-PD-1 therapy (68). Under the combination therapy of pembrolizumab with TP, only a suppression of the MCP-1 concentration occurred in the LHSCC patients. Thus, pembrolizumab can inhibit MCP-1 release in addition to the administration of TP alone. Suppression of MCP-1 showed overall positive results in terms of progression-free survival (PFS) and stimulation proved to be negative for patient survival. An increase in MCP-1 concentration during TP treatment may lead to tumor progression. These results are consistent with a more extensive study in mice with an intravenous CCR2-depleting antibody injection, which showed that depletion of CCR2+ monocytes in a therapeutic tumor setting leads to reduced tumor growth, demonstrating their immunosuppressive capacity. These results indicate that CCR2+ monocytes have a tumor-promoting effect, are counter-regulators of immunological tumor control and contribute to tumor evasion (90). The response to pembrolizumab vs. TP vs. pembrolizumab + TP suggests patient-specific effects of treatment on cytokine production *ex vivo*. The cytokines showing correlation with overall survival or even predicting patient outcome are the same as those found in previous studies *ex vivo* (55) and *in vivo* (59), but require validation in a cohort of patients treated with TP or pembrolizumab + TP, preferably in a randomized clinical trial, while simultaneously tested in FLAVINO using pembrolizumab, TP, and pembrolizumab + TP *ex vivo*. The checkpoint inhibitor used in this assay is pembrolizumab (Keytruda^®^, MK-3475, MSD Sharp & Dohme Corp, Whitehouse Station, USA), a humanized monoclonal antibody that targets and blocks the protein PD-1 (91). Pembrolizumab was already approved as monotherapy for the treatment of R/M HNSCC with prior platinum-containing chemotherapy. The approval is based in part on results from the KEYNOTE-012, KEYNOTE 040 trials (34, 36). Furthermore, binary and tertiary combinations with docetaxel (Taxotere^®^, Sanofi-Aventis) and cisplatin were tested. These two therapeutics already showed to dose-dependently reduce the viability and colony formation of HNSCC under laboratory conditions (64). Although efficacy of monoclonal antibodies in relapsed HNSCC has been noted in responders to therapy with consequent prolonged overall survival (OS), the therapy does not lead to the desired response in every patient, resulting in about one third of non-responders with minor (insufficient low) shrinkage of the tumor or disease progression (92). As non-responsiveness or even hyper-progression after pembrolizumab treatment are reported, we have to expect responders and non-responders to the therapy of pembrolizumab in combination with docetaxel and cisplatin as well. Subgroups of HNSCC that respond or do not respond to treatment need to be identified, as pembrolizumab is costly and non-responders who receive ineffective treatment have an increased risk of early recurrence, reduced quality of life and premature death. Patients who benefit from pembrolizumab despite PD-1 negativity also show need for investigation (38). As we were able to identify a subgroup of patients with adverse stimulation of MCP-1, IFN-γ and IL-6, and the increased levels of the three cytokines emerged as biomarkers for rather poor outcome, we reached the aim of this study by determining deviating effects of pembrolizumab, docetaxel and cisplatin on HNSCC and their cytokine release *ex vivo*. Monitoring these *ex-vivo* effects in parallel to cytokine measurements in blood samples during treatment and clinical follow-up in the ELOS trial will allow for assessing their value as biomarkers for successful pembrolizumab therapy. ELOS is a randomized, two-arm phase II study on organ preservation of the larynx in advanced laryngeal or hypopharyngeal squamous cell carcinoma (LHNSCC) in stage III, IVA/B, which are only resectable by total laryngectomy and have PD-L1 expression with CPS ≥ 1. This study is based on the two studies KEYNOTE-048 and DELOS-II (German Laryngeal Organ Preservation Study II). Positive results were seen in the KEYNOTE-048 trial as a first-line therapy for recurrent and metastatic HNSCC, as well as in the curative setting ADRISK (ClinicalTrials.gov NCT03480672) and NadiHN (EudraCT No. 2016-004787-20). The endpoints of the KEYNOTE-048 study were overall survival and progression-free survival in the intent-to-treat population. The results were positive in terms of efficacy and safety. Therefore, it can be concluded that pembrolizumab plus platinum and 5-fluorouracil is an appropriate first-line therapy for recurrent or metastatic HNSCC, and that pembrolizumab monotherapy is an appropriate first-line therapy for PD-L1-positive recurrent or metastatic HNSCC (9, 93). The randomized phase II DELOS-II trial investigated the effect of adding cetuximab to an already well-functioning therapy with TPF or TP and radiotherapy, also with the hope of laryngeal preservation in LHSCC (NCT00508664 (2–6)). Although cetuximab did not lead to any significant difference in the test group, it was positively noted that the standard group showed an unexpectedly positive development (93). Hence, TP is used for reference (control arm) also in the ongoing ELOS RCT (7, 8). Combination therapy of pembrolizumab in addition to docetaxel with cisplatin in patients with LHSCC identifies *ex vivo* those patients with prolonged PFS irrespective their treatment. Thus, we interpret this finding as an expression of a proper working immune system with potential to eradicate the tumor, provided the tolerance-inducing PD-1:PD-L1 immune-checkpoint can be blocked, for instance using pembrolizumab. This means that addition of pembrolizumab has a potential in these patients to overcome the immunosuppressive cancer microenvironment and may increase the frequency of responders (overall response rate), best response rate, OS, DSS, PFS, EFS and LFS. Further studies are needed to confirm the results and to identify the underlying mechanisms to realize the full potential of specific cancer immunotherapies. In addition to enhancing tumor-specific cytotoxic T cell responses, future immunotherapeutic approaches may need to focus on the immunosuppressive TME, including the role of CCR2+ monocytes, and the interplay with IFN-γ and IL-6 in HNSCC and the patients’ blood.

## Conclusions

Response evaluation of HNSCC treated *ex vivo* might allow for identification of responsiveness of an individual patient’s tumor to combination treatment with pembrolizumab + TP before starting induction chemotherapy. Measuring at least three cytokines, MCP-1, IFN-γ and IL-6 may be able to get very desirable information about principal responsiveness of the tumor to this treatment and, provided suppressed production of these cytokines, predict superior outcome.

## Data Availability

The raw data supporting the conclusions of this article will be made available by the authors, without undue reservation.
