# Supplementary figures and images for: Suppression of MCP-1, IFN-γ and IL-6 production of HNSCC ex vivo by pembrolizumab added to docetaxel and cisplatin (TP) exceeding those of TP alone is linked to improved survival
Source: Front Immunol. 2025 Jan 15;15:1473897. doi: 10.3389/fimmu.2024.1473897 (PMC11774711; doi:10.3389/fimmu.2024.1473897)

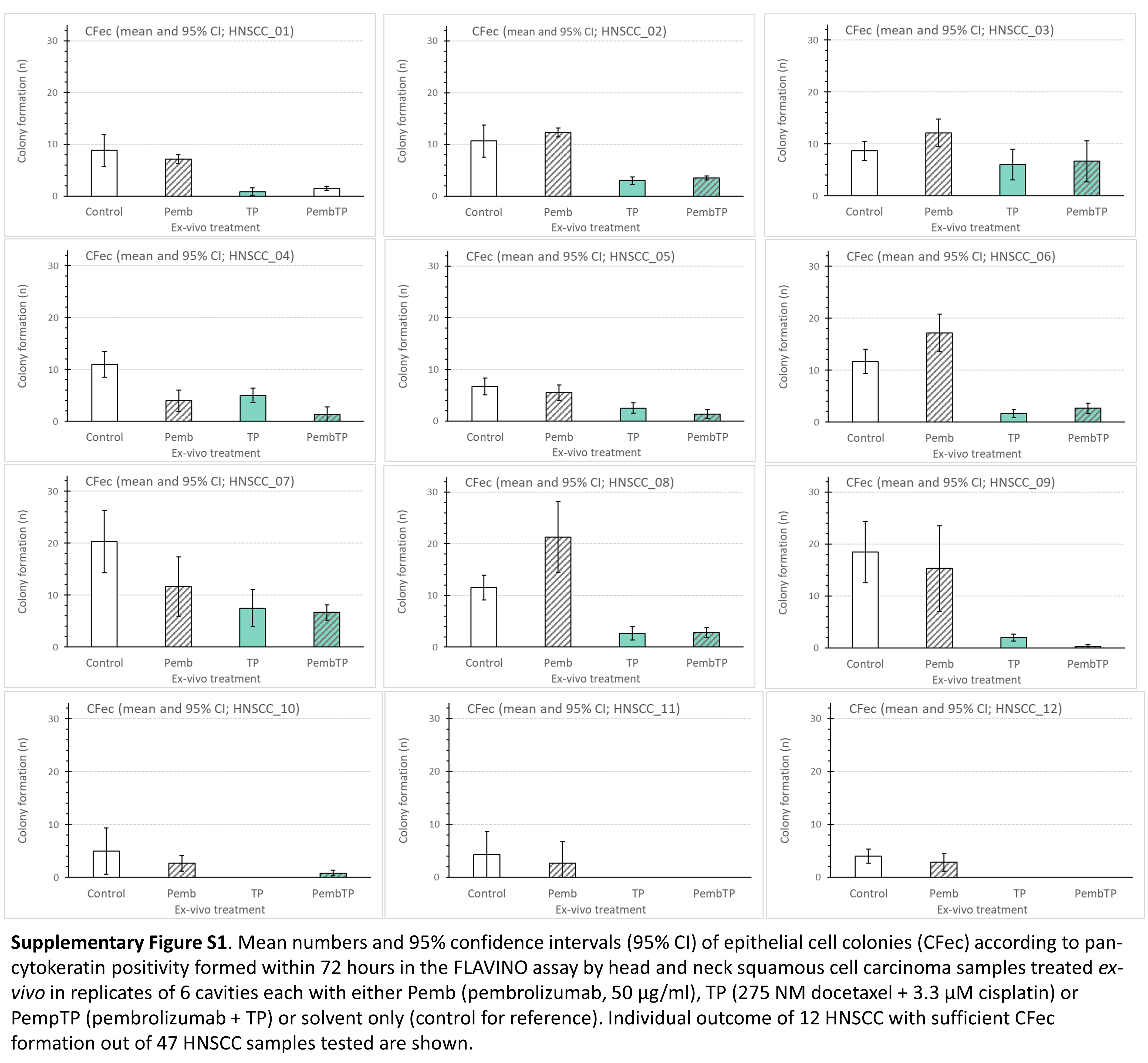

Supplement: Supplementary file 1 [file Image1.tif]
