# Supplementary material for: Suppression of MCP-1, IFN-γ and IL-6 production of HNSCC ex vivo by pembrolizumab added to docetaxel and cisplatin (TP) exceeding those of TP alone is linked to improved survival
Source: Front Immunol. 2025 Jan 15;15:1473897. doi: 10.3389/fimmu.2024.1473897 (PMC11774711; doi:10.3389/fimmu.2024.1473897)
Supplement: Supplementary file 2 [file Table1.pdf]

Supplementary Table S1. Statistical analysis of data shwon in Figure 1. Student's t tests for paired samples and groups of samples with insignificant differing variance (homoscedastic p values) and significant different variance according to the F test (heteroscedastic p values) are shown together with correlation according to Pearson's correlation coefficient r and the coefficient of determination R and the interpretation of the impact of either pembrolizumab or TP added as well as the interpretation regarding the direction of response. While most readouts according to efficacy were found to be homogenous, IFN-γ, IL-6 and MCP-1 demonstrated heterogeneity in responses to pembrolizumab alone or if combined with TP.

		***P* values (2-sided)**			**Correlation**			**Increased efficacy through**		
	**Treatment**	***t* test paired**	***t* test homo-scedastic**	***t* test hetero-scedastic**	***Pearson's r***	***R***	**Variance explained**	**Pemb**	**TP**	**Response**
**IP-10**	**Control vs. Pemb**	.10589	.52303	.52305	.854	.729	72.9%	insignificant	--	
	**Control vs. TP**	**.02073**	.0546	.05666	.593	.352	35.2%	--	significant	homogenous
	**Control vs. Pemb + TP**	**.01935**	**.02862**	**.03057**	.338	.114	11.4%	significant	significant	homogenous
	**TP vs. PembTP**	.32398	.53339	.53347	.623	.389	38.9%	insignificant	--	
	**Pemb vs. PembTP**	.21283	.24573	.24819	.340	.116	11.6%	--	insignificant	
**TNF-α**	**Control vs. Pemb**	.10092	.19372	.19453	.448	.201	20.1%	insignificant	--	
	**Control vs. TP**	**.00094**	**.03588**	**.03612**	.667	.445	44.5%	--	significant	homogenous
	**Control vs. Pemb + TP**	**.00409**	**.00487**	**.00538**	.124	.015	1.5%	significant	significant	homogenous
	**TP vs. PembTP**	.54649	.57139	.57176	.147	.022	2.2%	insignificant	--	
	**Pemb vs. PembTP**	**.00216**	**.02573**	**.02597**	.542	.294	29.4%	--	significant	homogenous
**IFN-γ**	**Control vs. Pemb**	.06054	.62476	.62478	.941	.886	88.6%	insignificant	--	**heterogeneous**
	**Control vs. TP**	.52316	.83511	.83517	.962	.926	92.6%	--	insignificant	
	**Control vs. Pemb + TP**	.56820	.83070	.83079	.952	.907	90.7%	insignificant	insignificant	
	**TP vs. PembTP**	.91344	.98914	.98914	.987	.974	97.4%	insignificant	--	
	**Pemb vs. PembTP**	.15664	.56104	.56142	.977	.955	95.5%	--	insignificant	
**IL-6**	**Control vs. Pemb**	.78609	.93371	.93372	.923	.852	85.2%	insignificant	--	
	**Control vs. TP**	.8189	.9097	.90974	.826	.682	68.2%	--	insignificant	
	**Control vs. Pemb + TP**	.06695	.42194	.42198	.822	.675	67.5%	insignificant	insignificant	**heterogeneous**
	**TP vs. PembTP**	.38147	.6244	.62469	.798	.637	63.7%	insignificant	--	
	**Pemb vs. PembTP**	**.02988**	.33013	.33014	.811	.658	65.8%	--	significant	**heterogeneous**
**IL-8**	**Control vs. Pemb**	.20593	.23234	.23496	.319	.101	10.1%	insignificant	--	
	**Control vs. TP**	.08876	.09648	.0994	.18	.032	3.2%	--	insignificant	
	**Control vs. Pemb + TP**	.72194	.86377	.86386	.884	.781	78.1%	insignificant	insignificant	
	**TP vs. PembTP**	.23625	.2376	.24049	.084	.007	0.7%	insignificant	--	
	**Pemb vs. PembTP**	.35925	.36642	.36865	.172	.03	3.0%	--	insignificant	
**MCP-1**	**Control vs. Pemb**	.07312	.64974	.64975	.944	.891	89.1%	insignificant	--	**heterogeneous**
	**Control vs. TP**	**.00005**	**.00388**	**.00441**	.873	.763	76.3%	--	significant	homogenous
	**Control vs. Pemb + TP**	**.00009**	**.00629**	**.00685**	.787	.619	61.9%	significant	significant	homogenous
	**TP vs. PembTP**	.47828	.85042	.85043	.943	.89	89.0%	insignificant	--	
	**Pemb vs. PembTP**	**.00050**	**.01616**	**.01691**	.728	.531	53.1%	--	significant	homogenous
**VEGF**	**Control vs. Pemb**	.64064	.7723	.7723	.619	.383	38.3%	insignificant	--	
	**Control vs. TP**	**.00001**	**.00004**	**.00006**	.339	.115	11.5%	--	significant	homogenous
	**Control vs. Pemb + TP**	**.00028**	**.00085**	**.0009**	.248	.061	6.1%	significant	significant	homogenous
	**TP vs. PembTP**	.37737	.45988	.46006	.323	.104	10.4%	insignificant	--	
	**Pemb vs. PembTP**	**.00236**	**.00208**	**.00215**	.033	.001	0.1%	--	significant	homogenous

Pemb - pembrolizumab (50 µg/ml); TP - docetaxel (275 nM) + cisplatin (3.3 µM); PembTP - pembrolizumab + TP; *R* - coefficient of determinaton $R = r^2$.
